# Apocrine adenocarcinoma of the head and neck district: Our experience with two cases

**DOI:** 10.1016/j.ijscr.2023.109017

**Published:** 2023-11-04

**Authors:** Francesco Ferragina, Ida Barca, Alfonso Sorrentino, Angelo Ruggero Sottile, Maria Giulia Cristofaro

**Affiliations:** a Department of Experimental and Clinical Medicine, Unit of Maxillofacial Surgery, “Magna Graecia” University, Viale Europa, 88100 Catanzaro, Italy; b Department of Neurosciences, Reproductive and Odontostomatological Sciences, Unit of Maxillofacial Surgery, University Hospital of Naples “Federico II”, 80131 Napoli, Italy

**Keywords:** Apocrine adenocarcinoma, Head and neck Cancer, Maxillofacial surgery, Oncological surgery

## Abstract

**Introduction:**

Apocrine adenocarcinoma (AA) is a rare gland cancer that appears in the elderly, especially males. Surgery is considered the first option for the management of this tumor.

**Case presentation:**

We report two cases of AA that occurred at our Unit of Maxillofacial Surgery. Precisely a case of a woman with AA with a usual presence at the eyelid level and a case of a man with AA with an unusual presence at the neck level.

**Discussion:**

This cancer generally arises in some specific areas of the body that present high concentrations of apocrine glands (such as in Case No.2). But it can also occur in less typical areas, such as the neck (such as in Case No.1).

**Conclusion:**

We discuss the surgical management of our cases: both based on our experience and literature data, we recommend extensive surgical excision.

## Highlights

• Apocrine adenocarcinoma is a rare tumour that arises from apocrine glands, that presents an increased incidence among older males.
• The anatomical regions most affected by Apocrine adenocarcinoma, as they have a higher concentration of this specific glandular type, are the armpits, the ear canal, the eyelid region, and the perianal and periumbilical regions.
• Although the data in the literature are scarce and do not allow us to make unambiguous recommendations for proper surgical management of Apocrine adenocarcinoma, it seems reasonable to recommend extensive excision of the neoplasm.

## 1 Introduction

Apocrine adenocarcinoma (AA) is a rare tumor that arises from apocrine glands; it presents an increased incidence among older males [1,2]. The apocrine glands are a subtype of exocrine glands located at the lower dermis junction and the subcutaneous fat [3]. Their duct is formed by a single layer of secretory cells presenting a pathognomonic secretion type: secretion occurs by decapitation of part of the apical cytoplasm of glandular cells [4]. This process involves 3 distinct phases. First, the apical cap is formed. This is followed by the formation of a dividing membrane at the base of the apical cap. Finally, tubules form parallel to the dividing membrane, which creates both a base for the secreted apical cap, as well as a roof for the remaining secretory cell.

These glandular cells present an eosinophilic cytoplasm and are surrounded by an external layer of myoepithelial cells that helps in the secretory process. Histologically, apocrine glands can be viewed using light microscopy with hematoxylin and eosin staining. Apocrine glands are nonfunctional before puberty, at which time they grow and commence secretion. Some apocrine glands have specific names, for example, those on the eyelids are referred to as Moll's glands, and those on the external auditory meatus are termed ceruminous glands. While they can be found in many locations on the body, they secrete specific products at each distinct location. They secrete a specific product which depends on their localization. Although the exact function of apocrine glands varies depending on the gland's location, apocrine glands are believed to be an evolutionary remnant of an odorous organ of animals. For example, the scent glands of the skunk are modified apocrine-type structures. The anatomical regions most affected by AA, as they have a higher concentration of this specific glandular type, are the armpits, the ear canal, the eyelid region, and the perianal and periumbilical regions [5,6]. It is very infrequent to find AA in other body regions, such as the face skin [7]. In the literature, there are few studies on AA at the level of the neck and on its surgical management. Below, we present two cases of unusual localization of AAs treated with surgery. The work has been reported in line with the PROCESS criteria [8].

## 2 Case series presentation

### 2.1 Case no.1

**Fig. 1:**
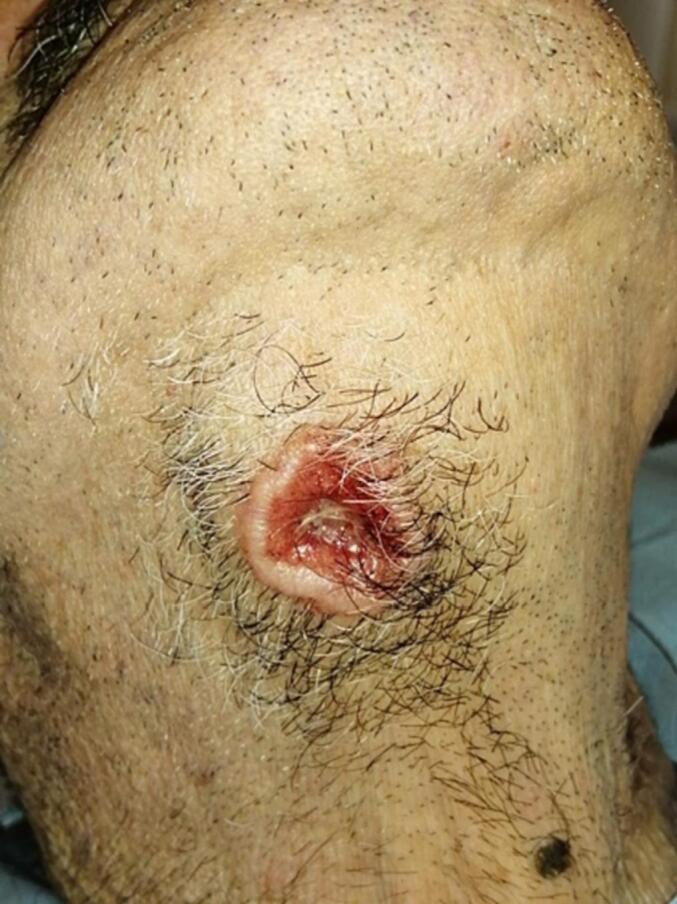
Clinical presentation of AA, large nonbleeding skin ulcer, with jagged margins, detected, and increased consistency.

A 77-year-old white man presented to the Maxillofacial Unit for a specialist surgical consultation; he presented an itchy nodule on the anterior surface of the neck, which had increased in size in the last six months. The patient's medical history was positive for arterial hypertension and chronic renal failure. Moreover, he did not present a personal or a family history of cancer. On physical examination, the patient was in poor health. It had a heteroplasic nodule of about 3.0 × 2.2 cm in diameter, with a large central ulcer with jagged and detected margins (Fig. 1).

**Fig. 2:**
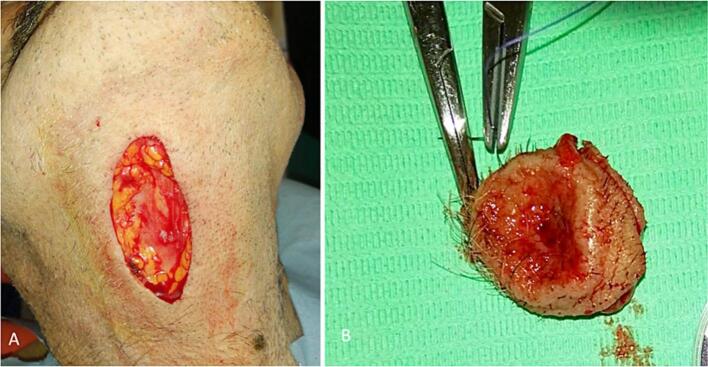
A) Intraoperative vision of AA removal. The wide margins of resection at the level of the subcutaneous fat and the underlying platysma muscle are highlighted. B) Anatomical specimen of apocrine adenocarcinoma of the neck.

There was no active bleeding. The tumor was surrounded by pilosebaceous units and presented with a solid texture without associated pain. The patient reported the appearance of a single nodule, grown over time, and underwent topical therapy based on cortisone and antibiotics. Such treatment had, however, led to a generalized irritation of the skin with the subsequent formation of a non-bleeding ulcer. Due to the patient's clinical condition (dialysis and bedridden), only an ultrasound of the neck regions was performed, negative for cervical lymphadenopathies. It showed the presence of poorly defined margins neoformation in the subcutaneous fat. Due to the lesion's ambiguous characteristics and the patient's poor physical condition, an excisional biopsy of the neoformation was chosen (Fig. 2).

**Fig. 3:**
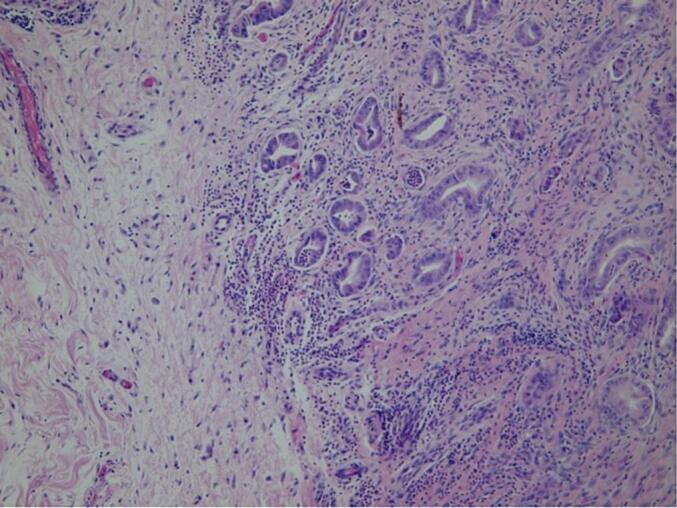
Invasive component of AA with staining with hematoxylin and eosin.

Excision was performed by ensuring about 1 cm of margins around the neoformation (the underlying platysma muscle has been displayed) to prevent margin infiltration in case of a malignant pattern. A primary wound closure was performed, with no other reconstructive techniques. Due to the poor patient's state of health, other more invasive surgical methods on the neck were not performed. Histopathological examination revealed the presence of AA infiltrating the dermis and infiltrating the hypodermis (EMA+, CAM 5.2+, Cytokeratin 5–6+) (Fig. 3).

All resection margins were free of neoplasia. The patient underwent periodic follow-up. After about 50 days he returned to check presenting a nodular neoformation during a scar from previous surgery. The nodule had an irregular morphology, poorly defined margins, a diameter of about 2 × 1 cm, a hard consistency, and pain. The patient underwent surgery again: the nodule was removed with an abundant portion of the subcutaneous fat, releasing the underlying muscular plane, which appeared macroscopically free from disease. Due to the abundant laxity of the neck tissues, a closure of the site was performed for first intention. Histopathological examination showed a Dermo-hypodermic recurrence of AA with ductal-papillary differentiation. Excision margins were free from neoplasia.

Unfortunately, due to the patient's poor clinical condition and worsening kidney disease, the patient died about 1 month after the second surgery.

### 2.2 Case no.2

**Fig. 4:**
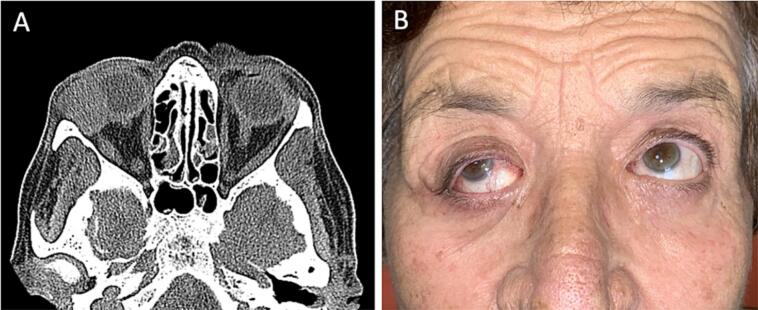
A) Computed Tomography: axial image showing a heterogeneous hyperintense mass, close to the lateral rectus muscle with inferior medial dislocation of the right eyeball. B) Preoperative view, showing ectropion with inferior medial dislocation of the right eyeball.

A 75-year-old white woman presented to the Maxillofacial Unit for a specialist surgical consultation; she presented a recurrence of a right orbit tumor. The patient was treated in 2018, in another hospital, for the surgical removal of an AA close to the external eye chant of the right eye. The patient underwent regular clinical follow-ups. After 6 months she noticed a neoformation at the orbital level, with a progressive increase in size in the following months. The volumetric increase was rapid and enough to dislocate the eye below (Fig. 4B).

**Fig. 5:**
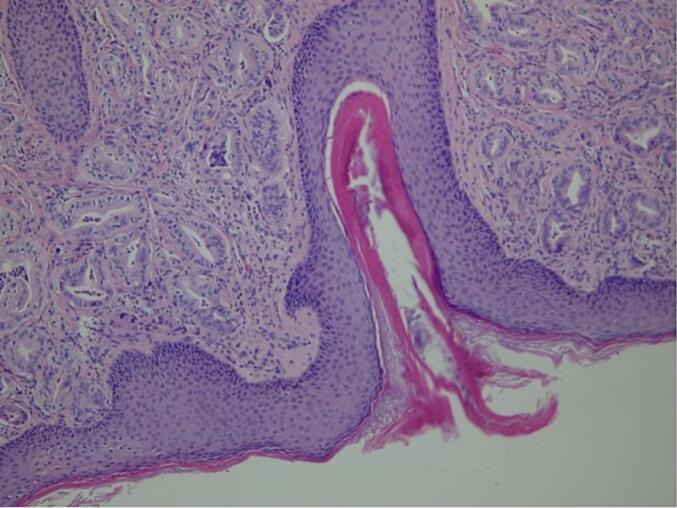
High lesion power showing AA lobular architecture and secretion by decapitation.

It was suggested to perform a computed tomography (CT) with a contrast medium. It showed the presence of a 22 × 18 mm heteroplasia that occupied the lateral portion of the right orbit, with oval morphology, and regular margins. It was contiguous with the right lateral rectus muscle and medially dislocated the eyeball, without invading it (Fig. 4A). The test was also negative for lymph node involvement. The surgical management of this case has been oriented only to the removal of the tumor without lymphadenectomy and with preservation of the orbital structures (respecting the patient's desire not to be subjected to invasive treatments on the eye). The tumor was approached with a superior-lateral skin incision (at the level of the one-third lateral of the right eyebrow arch). After the soft tissue dissection and the surgical exposure of the cortical bone, we tried to remove the neoplasm from the lateral face of the right orbit with blunt surgical instruments. The tumor was closely attached to the bone surface, which showed superficial erosion. We decided to perform an ostectomy of the upper orbital frame. Histopathological examination showed the presence of an AA (EMA+, CAM 5.2+, and Cytokeratin 5–6+) (Fig. 5).

An oncological and radiotherapy consultation was requested; no indication was given for adjuvant treatments. The patient carries out periodic clinical and instrumental follow-ups. At thirty-three months after surgery, the patient is free from disease.

## 3 Discussion

AA is a rare malignant neoplasm that affects the apocrine glands. It affects the elderly population, with an average age of 67 years, with a mild prevalence in male sex, and without any racial preference [9]. AA is typical of some anatomical areas (armpits, eyelids, ear canal, perianal and periumbilical regions), while it is rare at the level of the skin of the face and neck [10]. AAs can present different clinical behaviors, ranging from indolent models to more aggressive ones [11]. Their severity depends on their degree of differentiation. This tumor may also be localized or metastatic. In literature, the incidence of metastases occurs from 30 % to 50 % of cases and may present an invasion of lymph nodes [12,13]. Very often, especially in the most indolent forms, it can be erroneously diagnosed with a benign injury. The differential diagnosis is made with both benign and malignant tumors and metastases [13]. In the literature, as in a proposed case (No.1), skin irritation with subsequent ulceration after using ointments is also described [14]. To date, there are no specific guidelines for the management of AA. Surgical excision remains the first therapeutic option. Large local excision (extending 1–2 cm beyond the macroscopic end of the tumor) is always suggested [15]. Neck dissection should be done if there is evidence of lymph node involvement. Prophylactic neck dissection is controversial. There seems to be no evidence of benefit [16]. Some other studies suggest the importance of sentinel node evaluation in deciding the most appropriate intraoperative therapeutic choice [17]. It is recommended in case of unclear lymph node involvement because it may identify the clinically hidden disease [6,9,18]. Surgery may be associated with adjuvant treatments, such as radiation therapy and/or chemotherapy. They are used when lymph node involvement is not well defined or when lymph nodes are positive, in the case of advanced stages of pathology or G3/G4 classification [11,14]. The role of chemotherapy is unclear because AA is considered by some authors to be resistant [19]. Radiotherapy is recommended as a valid treatment both in the primitive forms and in the recurrences. It should be used when the resection margins are positive, in the case of high-classification tumors, and especially in the case of vascular and/or lymphatic invasion [14,20]. The absence of metastasis and lymph node involvement provides a better survival rate. Lymph node involvement is the parameter that best defines the prognosis [9].

## 4 Conclusion

Although the data in the literature are scarce and do not allow us to make unambiguous recommendations for proper surgical management of AA, it seems reasonable to recommend extensive excision of the neoplasm. Other therapeutic strategies can be performed depending on the location, size, stage, and lymph node involvement, from lymphadenectomy to medical treatment with chemotherapy/radiation therapy.

## Consent

Informed consent was obtained from the patient for presentation of the details of this case, along with the images for the purposes of publication. No personal identification information has been displayed in the images. A copy of the written consent is available for review by the Editor-in-Chief of this journal on request.

## Ethical approval

The study was conducted following the Declaration of Helsinki; the Magna Graecia University's Ethics Committee approved the study.

## Funding

This research received no external funding.

## Author contribution

**Conceptualization**: Cristofaro Maria Giulia, Ferragina Francesco, Barca Ida.

**Data curation**: Ferragina Francesco, Barca Ida, Sorrentino Alfonso, Cristofaro Maria Giulia.

**Formal Analysis**: Ferragina Francesco, Sorrentino Alfonso.

**Funding acquisition**: Sottile Angelo Ruggero.

**Investigation**: Ferragina Francesco, Barca Ida, Sorrentino Alfonso, Sottile Angelo Ruggero.

**Methodology**: Ferragina Francesco, Barca Ida, Sorrentino Alfonso.

**Project administration**: Cristofaro Maria Giulia, Barca Ida.

**Resources**: Ferragina Francesco, Sorrentino Alfonso, Sottile Angelo Ruggero.

**Software**: Sottile Angelo Ruggero.

**Supervision**: Cristofaro Maria Giulia.

**Validation**: Cristofaro Maria Giulia.

**Visualization**: Ferragina Francesco, Barca Ida, Sorrentino Alfonso, Cristofaro Maria Giulia.

**Writing – original draft**: Ferragina Francesco, Sorrentino Alfonso, Sottile Angelo Ruggero.

**Writing – review & editing**: Ferragina Francesco, Cristofaro Maria Giulia.

## Guarantor

Dr. Cristofaro Maria Giulia

## Conflict of interest statement

The authors declare that they have no conflict of interest.
